# Current Status of Nutritional Guidance for Pregnant Women in Japan: A National Survey

**DOI:** 10.31662/jmaj.2025-0288

**Published:** 2025-12-05

**Authors:** Nami Tamura, Jun Takeda, Naho Morisaki, Atsuo Itakura

**Affiliations:** 1 Department of Obstetrics and Gynecology, Faculty of Medicine, Juntendo University, Tokyo, Japan; 2 Department of Social Medicine, National Center for Child Health and Development, Tokyo, Japan

**Keywords:** Nutritional guidance, pregnant women, national survey

## Introduction

Nutritional management and appropriate gestational weight gain (GWG) are essential for maternal and child health. Inadequate nutrition increases risks of low birth weight (LBW), and preterm birth ^(1)^. In Japan, the average birthweight has declined over recent decades ^(2)^, raising concerns about later metabolic risk ^(3)^. In 2021, the Ministry of Health, Labour and Welfare updated the Dietary Guidelines for Pregnant and Lactating Women with higher GWG targets ^(4), (5)^, and in 2023, the Japan Society of Obstetrics and Gynecology (JSOG) incorporated these recommendations into clinical practice guidelines ^(6)^. Implementing these updates requires a multidisciplinary approach, including obstetricians, midwives, and dietitians. Dietitian-led counseling improves GWG control and reduces adverse outcomes such as LBW, preterm birth, macrosomia, gestational diabetes mellitus (GDM), and large-for-gestational age ^(7), (8)^. However, the extent to which nutritional counseling is delivered in practice and the degree to which the updated guidelines have been adopted across facilities remain unclear. We therefore assessed the current status of antenatal nutrition care in Japan, including adoption of the GWG guidelines and awareness of the dietary guidelines.

## Materials and Methods

### Study design

A nationwide cross-sectional questionnaire survey was conducted to evaluate the current status of nutritional guidance during pregnancy in Japan. A total of 3,804 delivery facilities, including comprehensive and regional perinatal centers, general hospitals, and clinics, were invited to participate.

### Survey distribution and data collection

Between December 18 and 31, 2023, postcards containing a QR code linked to a Google Form survey were sent to the directors of all participating facilities. The survey gathered information on the adoption of the 2021 GWG guidelines ^(5)^, awareness of the dietary guidelines ^(4)^, and the availability of registered dietitians for nutritional guidance. The questionnaire also inquired about the implementation status of outpatient nutritional guidance for obesity (body mass index [BMI] ≥30), iron-deficiency anemia, GDM, malnutrition, hypertensive disorder of pregnancy (HDP), excessive and insufficient weight gain during pregnancy, concern for macrosomia, and fetal growth restriction. For each condition, the responses were “Implemented for all”, “Implemented for those who wish”, “Not implemented”, or “No applicable pregnant women”. Only obesity, iron-deficiency anemia, GDM, malnutrition, and HDP are eligible for outpatient nutritional guidance reimbursement under the Japanese insurance system. Responses were collected, and the proportions of each response category were calculated.

### Statistical analysis

Descriptive statistics were used to summarize the responses. Chi-square tests were used to compare facility types according to whether they adopted the GWG guidelines or were aware of the dietary guidelines. Proportions were summarized with 95% confidence intervals (CIs) using the exact (Clopper-Pearson) method. Between-group differences were assessed using Pearson’s chi-square test with Yates continuity correction. These intervals quantify the statistical uncertainty due to sampling among respondents and do not adjust for potential non-response bias. All statistical analyses were performed using R software, and p < 0.05 was considered statistically significant.

## Results

**Figure 1.**
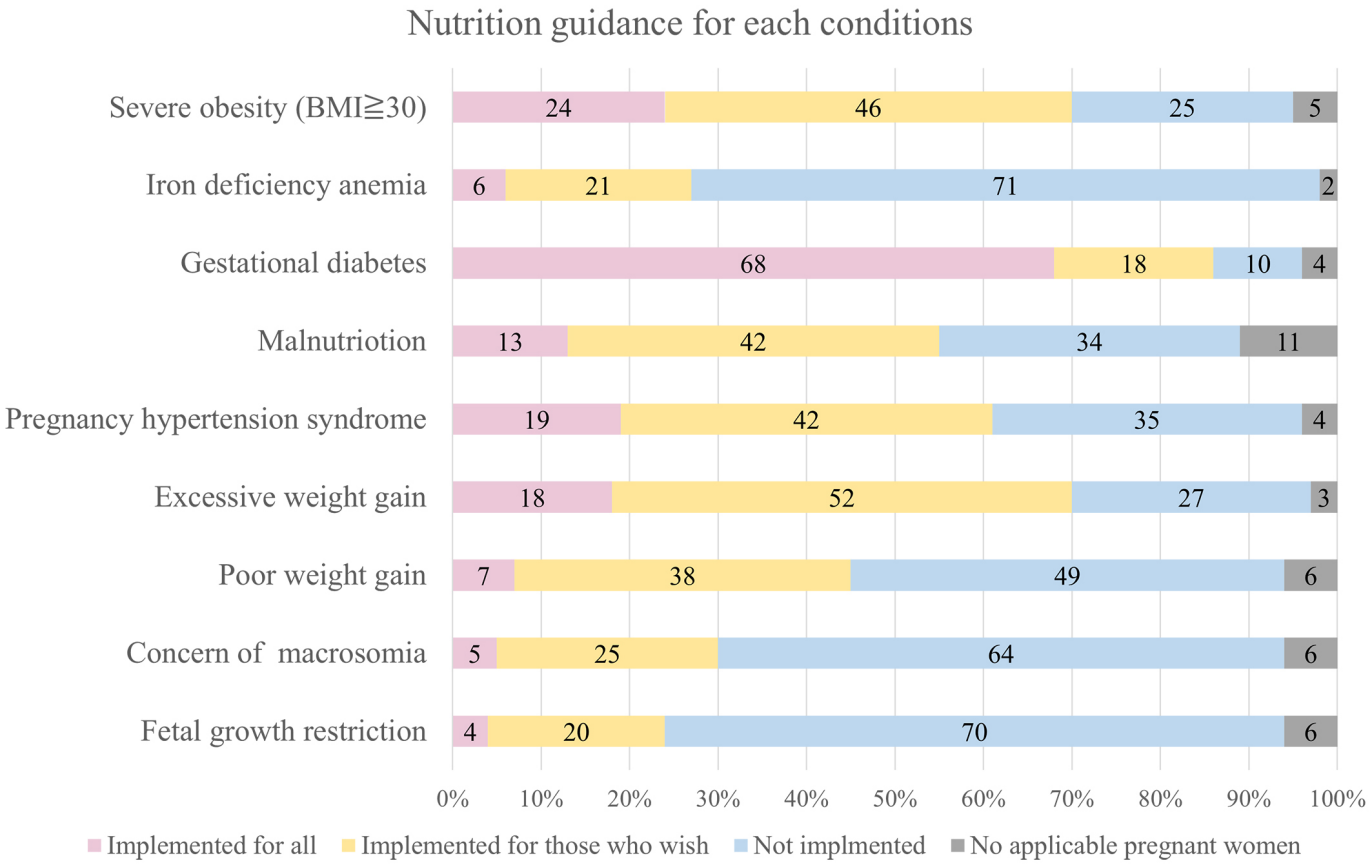
The figure shows the results of a national questionnaire survey on the provision of nutritional counseling according to maternal condition.

A total of 537 responses were received (response rate 14.1%). Of the responding facilities, 141 (19%) were comprehensive and regional perinatal centers, and 396 (81%) were general hospitals and clinics. Among the 537 surveyed facilities, 67.8% (95% CI: 63.7-71.8) reported adopting the GWG guidelines, and 63.6% (95% CI: 59.3-67.6) were aware of the dietary guidelines (Table 1). Regarding employed dietitians, 54.3% of facilities reported full-time employment, 6.7% reported part-time employment, and 39.0% reported no dietitian employment. Dieticians were employed by all perinatal centers and 51.7% of general hospitals and clinics. Among the 328 facilities with dietitians, only 23% provided routine nutritional counseling by registered dietitians to all pregnant women. Counseling was more commonly provided to women with obesity, GDM, and excessive weight gain. In contrast, counseling for the conditions of anemia and insufficient weight gain was less frequently implemented (Figure 1).

**Table 1.** Percentage of Awareness and Adoption of the 2021 Dietary and Gestational Weight Gain (GWG) Guidelines and Employment of Dietitians According to Facility Types.

		Total	Comprehensive/regional perinatal centers	General hospitals/clinics	p Value between facility types
Targeted facilities	n (%)	3,804	408 (10.7%)	3,396 (89.2%)	
Responding facilities	n (%)	538	103 (19%)	435 (81%)	
Response rate	%		25.2	12.8	< 0.001
Awareness of the 2021 Dietary Guidelines	% [95%Cl]	63.6 [59.3-67.6]	75.7 [66.3-83.6]	60.7 [55.9-65.3]	< 0.05
Percentage Adopting 2021 GWG Guidelines	% [95%Cl]	67.8 [63.7-71.8]	75.7 [66.3-83.6]	66.0 [61.3-70.4]	0.074
Employment of dietitians	(%)	60.6 [56.4-64.8]	100.0 [96.5-100.0]	51.4 [46.6-56.1]	< 0.001

p Values from Pearson’s χ^2^ test with Yates continuity correction; 95% CIs by exact binomial (Clopper-Pearson) CI: confidence interval.

## Discussion

This study reveals that the adoption of the 2021 GWG guidelines and awareness of dietary guidelines remain relatively low, especially in general hospitals and clinics, which constitute more than 70% of all obstetric facilities and where more than 70% of deliveries occur in Japan ^(9)^. Furthermore, the actual delivery of nutritional counseling varies significantly across facilities. Notably, guidance focused on reducing excessive intake was more commonly provided, whereas counseling for increasing the intake of deficient nutrients was less frequently provided.

No difference was observed in the adoption rate of the GWG guidelines according to facility type. This finding may be attributable to the fact that the GWG guidelines are incorporated into the clinical practice guidelines of the JSOG, which most obstetricians refer to in their routine clinical practice. In contrast, a significantly higher rate of awareness of dietary guidelines was observed in the perinatal center. Dietitians were employed in all perinatal centers, but only in 51.4% of general hospitals and clinics. A higher employment rate of dietitians in perinatal centers may increase multidisciplinary channels, such as conferences and antenatal classes, through which dietitians educate and affect obstetricians and midwives, promoting awareness and adoption of the dietary guidelines. The limited employment of registered dietitians restricts access to specialist nutrition services and may hinder comprehensive antenatal nutrition care, despite evidence of benefit ^(10)^. Other barriers to providing nutritional guidance during pregnancy may include limited time, resources, and training among healthcare providers ^(11)^. To address these gaps, we may directly measure perceived barriers using a validated checklist and qualitative interviews.

This study found that healthcare providers focused more often on counseling to reduce excessive intake than on addressing nutritional deficiencies. This suggests that they tend to focus on reducing the risk of short-term pregnancy outcomes, such as macrosomia, HDP, and emergency cesarean, rather than the long-term health outcomes of the child, for example preterm birth and LBW. This emphasis may reflect concerns about avoiding emergency cesarean sections in general hospitals and clinics with more limited surgical capacity than perinatal centers. In contrast, the limited counseling on nutrient deficiencies suggests under-recognition of their importance. Pregnant women are at an increased risk of micronutrient deficiencies, which can cause pregnancy complications and developmental problems ^(12)^. Anemia resulting from these deficiencies further increases the risk of LBW, preterm birth, and other adverse outcomes ^(13)^. Effective prevention requires ensuring adequate energy and micronutrient intake ^(14)^. Thus, to improve neonatal outcomes, including reducing LBW, nutrition counseling for malnutrition and anemia must accompany guidance on appropriate GWG.

There are some limitations in our study. First, the overall response rate was low, especially in general hospitals and clinics; thus, the overall proportions of awareness, adoption, and dietitian availability may be even lower. Non-responder characteristics were unavailable; therefore, residual bias cannot be ruled out. Second, the data were self-reported and may be subject to recall and social desirability biases; responses were not independently verified.

In conclusion, this study highlights the low uptake of the 2021 GWG and dietary guidelines and the suboptimal provision of counseling, particularly for malnutrition and iron-deficiency anemia. Improving access to dietitians and promoting the implementation of guidelines are essential for better maternal and neonatal outcomes.

## Article Information

### Author Contributions

Nami Tamura contributed to data acquisition, data analysis, interpretation of the results, and drafted the original manuscript. Jun Takeda and Naho Morisaki contributed to study conceptualization and interpretation of the results, contributed to data analysis and critically revised the manuscript. Atsuo Itakura critically revised the manuscript and supervised the conduct of the study. All authors reviewed the draft and approved the final version of the manuscript for publication.

### Conflicts of Interest

None

### Disclaimer

Naho Morisaki is one of the Editors of JMA Journal and on the journal’s Editorial Staff. She was not involved in the editorial evaluation or decision to accept this article for publication at all.

### IRB Approval Code and Name of the Institution

Ethics Committee of Juntendo University Hospital (E2023-12).
